# Effects of neem extract on *Artemia franciscana*: Insights from high-throughput transcriptomics and phenotypic analysis

**DOI:** 10.1371/journal.pone.0348463

**Published:** 2026-08-11

**Authors:** Rodolfo Farlora, Paulina Bustos, Eugene M. Tine, Eduardo Jeria, Alex Eapen, Padmakumar Pillai, Jorge Pino, Donald I. Brown, Kathleen Whitlock

**Affiliations:** 1 Laboratorio de Biotecnología Acuática y Genómica Reproductiva (LABYGER), Instituto de Biología, Facultad de Ciencias, Universidad de Valparaíso, Valparaíso, Chile; 2 Centro de Investigación y Gestión de Recursos Naturales (CIGREN), Universidad de Valparaíso, Valparaíso, Chile; 3 Laboratorio de Microbiología Integrativa e Innovación Biotecnológica, Instituto de Biología, Facultad de Ciencias, Universidad de Valparaíso, Valparaíso, Chile; 4 Laboratorio de Neurogenética y Desarrollo, Instituto de Neurociencia, Facultad de Ciencias, Universidad de Valparaíso, Valparaíso, Chile; 5 Laboratorio de Genética e Inmunología Molecular, Instituto de Biología, Pontificia Universidad Católica de Valparaíso, Valparaíso, Chile; 6 Research and Development, Cargill, Inc., Wayzata, Minnesota, United States of America; 7 Laboratorio de Biología de la Reproducción y del Desarrollo, Instituto de Biología, Facultad de Ciencias, Universidad de Valparaíso, Valparaíso, Chile; 8 Centro Interdisciplinario de Neurociencias de Valparaíso (CINV), Universidad de Valparaíso, Valparaíso, Chile; VIT University, INDIA

## Abstract

Neem-derived biopesticides are increasingly applied in agriculture and have been tested in aquaculture research, yet their effects on non-target aquatic invertebrates remain insufficiently characterized. We evaluated the effect of neem extract on the brine shrimp *Artemia franciscana* using an integrated ecotoxicological approach combining phenotypic, transcriptomic, and histological analyses. Juvenile *A. franciscana* exhibited dose-dependent mortality and sublethal abnormalities, with a 24 h median lethal concentration of 292.48 mg/L (95% confidence interval, 257.75–331.89) for mortality and a median effective concentration of 146.36 mg/L (95% confidence interval, 113.04–189.50) for the combined endpoint “abnormal + dead”. In adults, males showed greater mortality than females after extended exposure. High-throughput RNA sequencing revealed broad treatment-associated differences in transcript abundance, with juveniles displaying downregulation of detoxification enzymes and chitin biosynthesis genes, alongside enrichment of immune- and cuticle-related gene ontologies. Adults showed transcriptional signatures of stress, including upregulation of heat shock proteins and cytoskeletal components, and suppression of genes involved in energy metabolism. Chitin precursor enzymes were selectively downregulated in males, and altered carbohydrate metabolism was observed in females. Histological analyses showed treatment-associated alterations in the embryo-bearing brood sac region and reduced ovarian area in treated females. Overall, neem exposure was associated with phenotypic, histological, and transcriptomic changes in *A. franciscana*. These results support the use of combined transcriptomic and histopathological endpoints to characterize responses to plant-derived biopesticides in aquatic arthropods.

## 1. Introduction

The neem tree (*Azadirachta indica*) produces bioactive compounds, primarily azadirachtins, widely employed as botanical insecticides [1,2]. Azadirachtin A, a major limonoid constituent of many neem-derived preparations, has been described as an antifeedant, insect growth-disrupting compound and sterilant, with effects on molting, metamorphosis, and endocrine-regulated developmental processes in insects [3]. Neem-derived formulations have also been tested experimentally in aquaculture settings, including neem leaf treatment against *Diplectanum* infection in European sea bass (*Dicentrarchus labrax*) [4], chitosan neem nanocapsules to enhance immunity and disease resistance in Nile tilapia (*Oreochromis niloticus*) [5], neem extract against sea lice (*Lepeophtheirus salmonis*) infestations in Atlantic salmon (*Salmo salar*) [6], and dietary neem leaf supplementation to modulate immune response and disease resistance in Asian seabass (*Lates calcarifer*) challenged with *Vibrio harveyi* [7]. However, the use of neem-based products in systems connected to natural or semi-natural water bodies raises concerns about potential adverse impacts on non-target aquatic organisms, particularly invertebrates that underpin food-web structure [8].

Transcriptomics has emerged as a valuable tool in ecotoxicological studies of aquatic species, offering insights into the molecular mechanisms underlying toxicant exposure and facilitating the development of sensitive molecular biomarkers [9–11]. High-throughput transcriptome sequencing (HTS) can provide broad surveys of gene expression changes associated with toxicant exposure, allowing the identification of candidate responsive genes and biological pathways [12,13]. Increasingly, such mechanistic information is organized within Adverse Outcome Pathway (AOP) frameworks, which link molecular and cellular key events to organism- and population-level adverse outcomes for use in ecotoxicological risk assessment [14,15].

The brine shrimp, *Artemia franciscana*, serves as a valuable model for comparative toxicology and invertebrate physiology, combining ease of culture and sensitivity to chemically induced physiological disruption [16–19]. In addition, *Artemia* nauplii are extensively used in marine fish and crustacean larviculture because cysts can be hatched on demand, nauplii are suitable prey for larval feeding, and they can be used as vehicles for nutritional enrichment [20]. Although acute toxicity and general physiological responses to various contaminants in the genus *Artemia* have been extensively investigated [21,22], mechanistic data describing how neem-derived compounds affect molecular pathways and tissues in this branchiopod remain limited, despite the growing use of azadirachtin-based neem biopesticides and evidence of adverse effects in other aquatic invertebrates [8,23]. To our knowledge, no previous study has combined transcriptomic and histological endpoints to investigate sublethal effects of neem exposure in *A. franciscana*.

To address this gap, we combined traditional phenotypic endpoints (survival and developmental abnormalities), RNA-seq transcriptomics and histological examinations to evaluate neem extract toxicity in juvenile and adult *A. franciscana*. Specifically, we (i) quantified dose-dependent mortality and developmental abnormalities in juveniles, (ii) characterized adult survival patterns and reproductive condition under subchronic exposure, (iii) identified life stage- and sex-specific transcriptomic responses to neem treatment, with a particular focus on chitin biosynthesis and cuticle-related pathways, and (iv) assessed histological alterations in adult reproductive tissues. Together, these data provide an integrated assessment of phenotypic, histological, and transcriptomic responses to neem exposure in this model crustacean, and identify candidate biological processes associated with treatment.

## 2. Materials and methods

### 2.1. Preparation of neem extract

Neem kernel extract stock solution (3 g/L) was prepared from a commercial product (Coromandel/Murugappa; Parry’s mBio NeemAzal® Technical Pack No 06/1/3, Lot No B365). This formulation contains azadirachtin A (25–50%) along with other naturally occurring kernel constituents, including additional azadirachtins (14–21%), limonoids (1–10%), and fatty acids (1–2%). These values correspond to the compositional range declared for the commercial product and were not independently quantified in the present study. Therefore, exposure concentrations are reported as nominal concentrations of total neem extract in mg/L or ppm, rather than as analytically measured concentrations of azadirachtin A or individual limonoids. Because of their low solubility in water, dilutions were made in 0.5% dimethyl sulfoxide (DMSO) in artificial seawater. The control group received artificial seawater containing 0.5% DMSO only, corresponding to the solvent concentration used in all neem extract dilutions. The stock was stirred overnight at room temperature before use. Neem concentrations are reported as ppm (mg/L).

### 2.2. Culture of juvenile and adult *Artemia*

*A. franciscana* were maintained in artificial seawater at 35–40 ppt and 28°C under a controlled 14 h light/10 h dark cycle. *A. franciscana* cysts were hatched under the same salinity, temperature, and photoperiod conditions. Juvenile *Artemia* were collected 12–24 h post-hatching, corresponding to nauplius instar stages I–II, and placed in 24-well plates with approximately 50 juveniles per well. Artificial seawater was removed, and 2 mL of test solution were added to each well. Test solutions consisted of nominal neem extract dilutions prepared in artificial seawater at 35–40 ppt with 0.5% dimethyl sulfoxide (DMSO), as described above. Control wells contained artificial seawater with 0.5% DMSO only. Nauplii were incubated at neem extract concentrations ranging from 0 to 800 ppm for 24 h at 28°C under a 14 h light/10 h dark cycle and scored after 24 h. Test solutions were not replaced during the 24 h juvenile exposure.

At nauplius instar stages I–II, normal individuals showed active swimming with coordinated undulating movements of the podia (S1 Video; control, 00:04–00:10) and were scored as normal. Individuals showing reduced and/or uncoordinated podial movements and impaired ability to remain in the water column were classified as abnormal (S1 Video; 00:17–00:30). Individuals that remained immobile at the bottom of the well, showed no response to gentle tapping, and exhibited necrotic morphology were classified as dead (S1 Video; 00:30–00:58). The results were expressed as lethal concentration (LC) values for mortality. The effective concentration (EC), representing the percentage of abnormal and deceased animals within the population, was also calculated.

Adult *A. franciscana* were grown and maintained as a laboratory colony in artificial seawater at 35–40 ppt and 28°C under a controlled 14 h light/10 h dark cycle (S2 Video). Adults used in the exposure assays were sexually mature individuals selected by external reproductive morphology: males were identified by the presence of claspers, and females were selected based on the presence of a visible brood sac. Experimental groups were maintained in six-well culture plates containing 10 mL of test solution per well, with three individuals of the same sex per well. Each concentration (0, 250, and 500 ppm) was tested in six replicate wells (n = 18 adults per sex and treatment). Animals were exposed to nominal neem extract dilutions prepared in artificial seawater at 35–40 ppt with 0.5% DMSO, using the same solvent concentration as in the juvenile assays. Adults were maintained for seven days under the same temperature and photoperiod conditions described above, with daily renewal of the full test solution volume (10 mL per well). Animals were fed daily with SERA® Micron Nature Fry Food (SERA GmbH), supplied as two drops per well from a 1 g/L suspension prepared in artificial seawater. Because no consistent sublethal abnormalities could be reliably scored in adults, mortality was recorded daily throughout the 7-day exposure period.

Overall, the experimental design comprised an acute 24 h juvenile exposure (0–800 ppm) used for toxicity assessment and juvenile RNA-seq in the sublethal range (0–400 ppm), and a 7-day adult exposure that supported three endpoints: survival analysis at 0, 250 and 500 ppm, adult RNA-seq at 0, 250 and 500 ppm, and histology assessments at 0, 100, 250 and 500 ppm.

### 2.3. Total RNA extraction and construction of cDNA libraries

After the 24-hour exposure to the sublethal range of neem extract (0 ppm control, 100, 200, 300 and 400 ppm), with each treatment performed in triplicate (three wells per concentration; approximately 50 juveniles per well), juveniles were collected from each well using filter paper, preserved in RNAlater (Thermo Fisher Scientific), and stored at −80 ºC. Each well was treated as one biological replicate, yielding three independent RNA samples per juvenile treatment.

Adult male and female *Artemia* were exposed for seven days to neem extract at 0 ppm (control), 250 ppm, and 500 ppm. For each sex and concentration, three RNA-seq libraries were prepared. Each library consisted of a pool of three surviving adults collected at day 7 from the same sex and treatment group. When necessary, individuals were combined from more than one well within the same sex and treatment group to obtain the number required for library construction.

Total RNA was extracted from pooled brine shrimp using the TRIzol reagent (Invitrogen) protocol. RNA purity was assessed using the A260/A280 ratio with a NanoDrop Lite spectrophotometer (Thermo Fisher Scientific), and RNA integrity was verified by visualizing samples in agarose gels under denaturing conditions. Subsequently, a total of 33 double-stranded cDNA libraries were generated using the TruSeq® RNA Sample Preparation Kit v2 (Illumina®) by Novogene USA, corresponding to three biological replicates per treatment and life stage/sex combination (five juvenile treatments × three replicates, and three adult treatments × two sexes × three replicates). All libraries were sequenced on the NovaSeq 6000 platform (Illumina®) with 2 × 150 bp paired-end reads. The list of sequenced cDNA libraries is detailed in S1 Table. The original sequencing files have been uploaded to NCBI’s Sequence Read Archive (SRA) under the BioProject ID PRJNA1402122.

### 2.4. Sequencing of cDNA libraries and bioinformatics analysis

Transcriptomic analyses were conducted as an exploratory transcript-level assessment of treatment-associated abundance patterns using a de novo assembled reference transcriptome generated from the RNA-seq libraries included in this study. De novo transcriptome assembly can provide a useful reference for differential abundance analyses in emerging or non-conventional model systems, particularly when the objective is to generate a transcript-level resource from the biological material under study [24]. After adapter removal and quality trimming, clean reads were assembled de novo into a unified transcriptome using CLC Genomics Workbench software v11.0.1 (CLC Bio). The assembly parameters included a mismatch cost of 2, an insert cost of 3, a minimum contig length of 400 bp, a similarity threshold of 0.9, and a trimming quality score of 0.05. This assembly yielded 88,367 contigs that were subsequently annotated to the NCBI non-redundant and UniProtKB/SwissProt Protein databases using the BLASTx tool. The de novo assembled transcriptome and the corresponding BLAST-based annotation table are publicly available in BioStudies under accession S-BSST3203. Because this analysis was based on a de novo transcriptome assembly, different contigs may represent fragmented transcripts, alternative isoforms, paralogous sequences, redundant assemblies of the same gene, or chimeric sequences [25]. Accordingly, expression results are interpreted at the transcript/contig level rather than as definitive gene-level estimates.

The annotated transcriptome served as a reference for differential expression analysis by RNA-seq. Clean sequences obtained from each library were aligned to the reference transcriptome using CLC Genomics Workbench software. Expression values were calculated as transcripts per million mapped reads (TPM). The complete juvenile and adult transcript-abundance datasets are publicly available in BioStudies under accession S-BSST3203. Differentially expressed contigs meeting the criteria of |log2FC| ≥ 4 and a false discovery rate (FDR) p-value < 0.05 were identified and visualized in hierarchical clustering heat maps. Hierarchical clustering employed Euclidean distance and full linkage as parameters for analysis.

Gene Ontology (GO) enrichment analysis was performed after differential expression analysis using the Rank-based Gene Ontology Analysis with Adaptive Clustering (RBGOA) tool [26]. GO categories related to molecular function, biological process, and cellular component were evaluated using the differential-expression results, including Log_2_ Fold Change (log2FC) and p-value information [27]. Only annotated sequences with an E-value < 0.01 against UniProt entries were retained for GO-based interpretation.

### 2.5. In silico identification of genes associated with chitin synthesis

To investigate chitin synthesis in *A. franciscana*, a gene panel was curated from the assembled transcriptome, selecting contigs associated with this process through bioinformatic analysis and literature search on the genes and pathways associated with chitin metabolism in various crustacean species [28]. Homology searches against the NR protein database (E-value < 0.005) identified sequences related to chitin synthesis. These gene panels were used as reference datasets and reads from larvae and adults across treatment replicates were mapped against them using CLC Genomics Workbench, applying the same RNA-seq settings and hierarchical clustering parameters as described previously. Expression levels for the selected genes were quantified as transcripts per million (TPM) derived from the mapped libraries, and these values were used for subsequent analyses.

### 2.6. Histological analysis of adult *A. franciscana*

A total of 112 adult *A. franciscana* were selected for histological evaluation (14 males and 14 females per concentration), and these individuals were reserved exclusively for histological processing. Animals were fixed in Bouin's solution for 48 h and subsequently rinsed under running tap water. Standard histological procedures were followed, including dehydration through a graded ethanol series, clearing in butanol, and embedding in Paraplast Plus® (Merck). Embedded tissues were sectioned at 5 μm thickness using a Leica RM2255 microtome and mounted on glass slides. For visualization, tissue sections were dewaxed and rehydrated via a xylene-butanol sequence and processed for trichrome staining: Hematoxylin (Merck) for nuclei, Erythrosin-orange G (Merck) for cytoplasmic differentiation, and Aniline blue (Merck) for connective tissues and secretion granules. Slides were dehydrated in ethanol, cleared in xylol (Merck), and mounted in Entellan™ (Merck). A Leitz-Leica DMRBE microscope equipped with a Leica DFC290 digital camera was used to document histological features.

The gonadal areas of male and female individuals were identified and photographed, with images taken from at least three consecutive sections per individual when available. Gonadal area measurements were performed from digital micrographs using ImageJ software (version 1.54h; National Institutes of Health) after calibration with the microscope scale bar. Areas were quantified from consecutive sections per individual and averaged for downstream analysis. Representative histological images, including EBS-associated alterations in treated females, are presented in the main figures, and additional quantitative data on gonadal area measurements are provided in the supplementary material.

### 2.7. Statistical analysis

Statistical analyses were conducted to evaluate the effects of neem extract exposure on *A. franciscana* at phenotypic, transcriptomic, and histological levels. Phenotypic data, including mortality and developmental abnormalities in juveniles, were analyzed using one-way analysis of variance (ANOVA), followed by Tukey’s multiple comparison test to determine significant differences between groups (*P* < 0.05). For adult survival analysis, a Kaplan-Meier survival plot was generated using GraphPad Prism software v10.4.1 (GraphPad Software), with statistical differences evaluated using the log-rank (Mantel-Cox) test in the same software.

For transcriptomic analysis, Principal Component Analysis (PCA) was performed on transcripts per million (TPM) values derived from paired-end cDNA libraries, projecting samples onto a two-dimensional space defined by the first and second principal components of the covariance matrix. Differential gene expression analysis was conducted using a T-test with false discovery rate (FDR) correction for multiple comparisons. Independent filtering was applied using a cutoff of |log2FC| ≥ 4 to minimize false positives, and a final subset of transcripts was retained for further analysis, with statistical significance set at *P* < 0.05. All RNA-seq statistical analyses were performed using CLC Genomics Workbench software.

For the expression analysis of chitin-related genes, TPM values were used as input for statistical comparisons, and gene expression differences in juveniles were assessed using one-way ANOVA, followed by Tukey’s multiple comparison test (*P* < 0.05). In adults, chitin-related gene expression was analyzed using two-way ANOVA, followed by Tukey’s multiple comparison test to evaluate differences between treatment groups (*P* < 0.05).

For histometric data, normality was assessed using the Shapiro–Wilk test. In cases where data did not meet normality assumptions, Box-Cox transformation was applied [29]. Statistical comparisons between experimental conditions were performed using one-way ANOVA, followed by Tukey’s multiple comparison test (*P* < 0.05).

All statistical analyses for phenotypic data, chitin-related gene expression, and histometric data were conducted using GraphPad Prism software (10.4.1).

### 2.8 Ethics statement

This study involved *Artemia franciscana*, a non-cephalopod invertebrate, and did not involve human participants, vertebrate animals, cephalopods, or field research. Therefore, institutional ethics approval was not required.

### 2.9. Artificial intelligence tools and technologies

During manuscript preparation, the authors used ChatGPT (OpenAI) to assist with language editing and to improve the clarity and consistency of the manuscript text. All AI-assisted output was reviewed and edited by the authors for accuracy and consistency with the original results and interpretations. No primary data, analyses, results, figures, tables, or Supporting Information files were generated by the AI tool. The authors take full responsibility for the content of the manuscript.

## 3. Results

### 3.1. Neem extract induces dose-dependent mortality and abnormal phenotypes in juvenile *A. franciscana*

At 24 h, all juveniles in the control group (0 ppm) exhibited a normal phenotype (Fig 1A). At concentrations below 100 ppm, most juveniles remained normal. Between 100 and 300 ppm, the proportion of abnormal juveniles increased significantly (P < 0.001), accompanied by a marked reduction in the proportion of normal individuals. At 200 ppm, only ~17% of juveniles remained normal, whereas abnormal and dead individuals accounted for the majority of the population. The proportion of abnormal juveniles peaked at 300 ppm (~64%) and then declined at higher concentrations as mortality became the predominant response. Mortality increased markedly from 400 ppm onward, exceeded 85% at 600 ppm, and reached approximately 95% at 800 ppm. Based on these concentration-response data, the 24 h LC₅₀ for juvenile mortality was 292.48 ppm (95% CI: 257.75–331.89), and the EC₅₀ for the combined endpoint “abnormal + dead” was 146.36 ppm. The replicate-level juvenile phenotype counts and percentages used to generate Fig 1A are provided in S1 Dataset.

**Fig 1:**
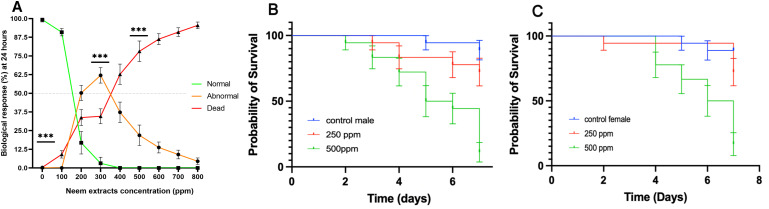
Dose-dependent effects of neem extract on survival and developmental phenotype of *Artemia franciscana.* **(A)** Juveniles exposed for 24 h. The proportion of individuals displaying a normal phenotype (green) decreased with increasing neem concentration, while abnormal phenotypes (orange) increased, peaking at 300 ppm, and declined at higher concentrations as mortality (red) increased. Data are shown as mean ± SD. Statistical differences were evaluated using one-way ANOVA followed by Tukey’s multiple comparison tests. **(B, C)** Survival of adult *Artemia*. Kaplan–Meier survival curves are shown for (B) males and (C) females exposed to 0, 250, and 500 ppm neem. Statistical differences were evaluated using the log-rank (Mantel–Cox) test.

### 3.2. Neem extract exposure reduces adult survival in *A. franciscana*

Preliminary observations indicated that adult *A. franciscana* were more resistant to neem extract than juveniles, which exhibited phenotypic effects within 24 hours of exposure. Therefore, the potential effects of neem extract on adults were assessed over a period of 7 days. Males and females were analyzed separately, with males identified by the presence of claspers, and only gravid females included in the study (S2 Video). At the lower concentration tested (250 ppm), some mortality was observed by the end of the 7-day exposure, but the difference from controls was not statistically significant. After seven days of exposure to 500 ppm, both males and females showed pronounced mortality (>80% in each sex; Fig 1B for males, Fig 1C for females), whereas control groups exhibited low mortality (approximately 11% by day 7, with deaths beginning on day 5). At both 250 and 500 ppm, male survival declined earlier than female survival in the plotted trajectories. During the exposure period, females in all groups continued to release free-swimming nauplii (ovoviviparous reproduction). The daily adult survival counts used to generate Fig 1B and Fig 1C are provided in S1 Dataset.

### 3.3. RNA-seq reveals widespread gene expression changes in neem-treated *A. franciscana*

To explore transcript-level responses associated with neem exposure, we performed a *de novo* high-throughput transcriptome assembly to serve as a reference for differential expression analysis. After quality filtering, the sequencing yielded 1,604,747,440 clean reads (**Table 1**), which assembled into 88,367 contigs with an N50 of 1,120 bp. About 70% of contigs (61,999 sequences) were successfully annotated against protein databases, which served as the reference for differential expression analysis.

**Table 1:** Summary of RNA-seq output and de novo transcriptome assembly statistics for *Artemia franciscana*. Values are based on 33 cDNA libraries from juveniles and adults and include total raw and clean reads, mean read length, number of contigs, total assembled bases, mean contig length, N50 and GC content.

Description	Statistics
**Total number of raw read sequences**	1,626,584,784
**Total number of clean read sequences**	1,604,747,440
**Mean length (bp)**	149.2
**Total number of contigs**	88,367
**Number of bases**	87,488,508
**Mean length of contig (bp)**	990
**N50 length of contig (bp)**	1,120
**GC% of contig**	40.03

The RNA-seq design included concentration-specific treatment groups. Juvenile libraries represented 0, 100, 200, 300, and 400 ppm neem extract, whereas adult libraries represented 0, 250, and 500 ppm neem extract in males and females. Across all samples, the exploratory screening identified a broad set of candidate treatment-responsive transcripts. Principal component analysis (PCA) of the global gene expression profiles (Fig 2) revealed a clear separation between juveniles and adults along the first principal component (Fig 2A). When analyzed within each life stage, control samples separated from neem-exposed samples. In juveniles, exposed groups separated from controls along PC1 (Fig 2B), with the 100 and 200 ppm groups clustering closer to each other and the 300 and 400 ppm groups showing partially overlapping but distinct profiles. In adults, transcript abundance profiles also diverged between control and exposed individuals (Fig 2C), while PC2 separated males from females, consistent with sex-specific transcriptomic differences in *Artemia*. Notably, the number of neem-responsive differentially-expressed contigs was much higher in adults (19,957) than in juveniles (933).

**Fig 2:**
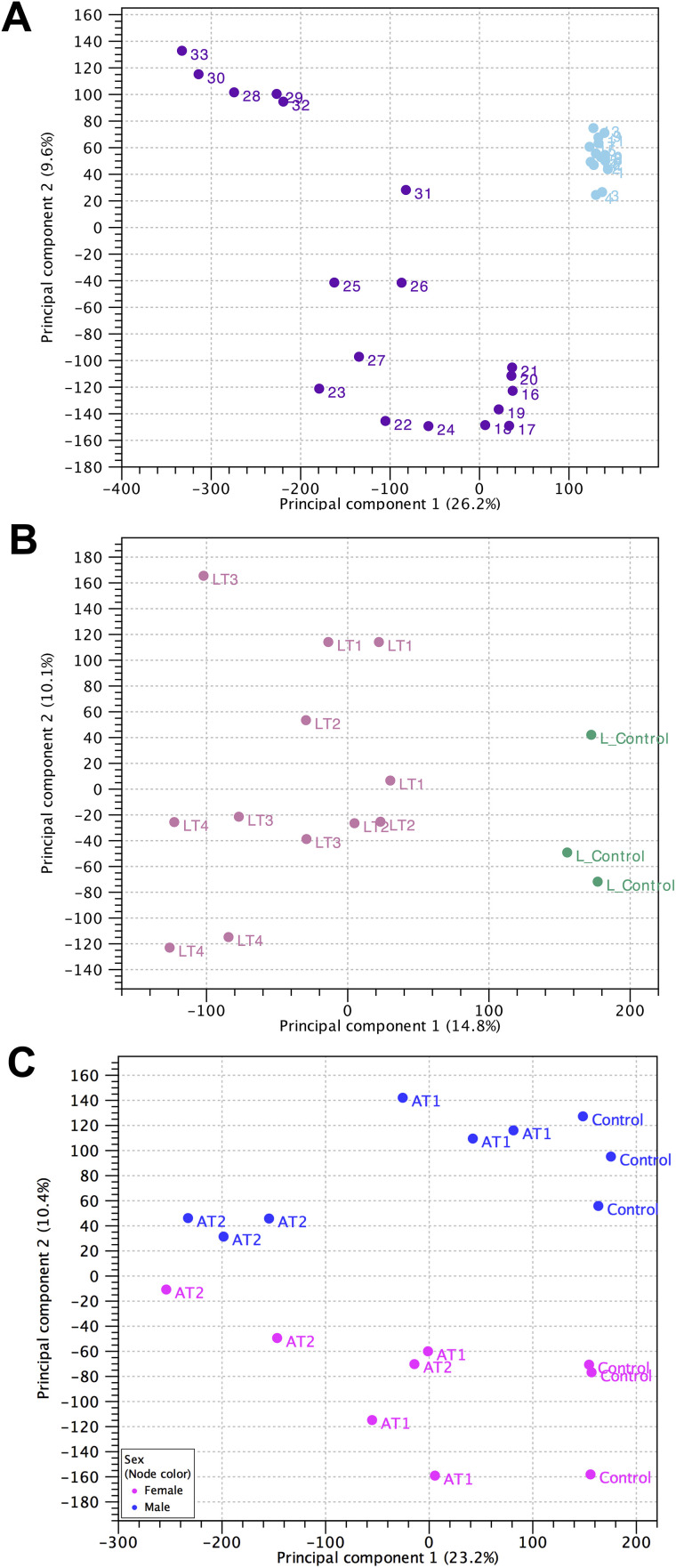
Principal component analysis (PCA) of global gene expression profiles in *A. franciscana* exposed to neem extracts. **(A)** PCA of all RNA-seq libraries (n = 33) showing separation between juveniles and adults along the first principal component (PC1). Each point represents one library; colours and/or symbols indicate life stage, sex and treatment (see panel legend). **(B)** PCA of juvenile libraries only (0, 100, 200, 300 and 400 ppm of neem extract), showing separation between control and neem-treated groups along PC1. **(C)** PCA of adult libraries only (0, 250 and 500 ppm of neem extract), illustrating differences between control and treated samples and separation between males and females.

### 3.4. Transcript abundance patterns in juvenile *A. franciscana* exposed to neem are consistent with metabolic and cuticle-associated candidate responses

Hierarchical clustering of differentially expressed contigs revealed distinct transcriptional differences between untreated and neem-treated juvenile *A. franciscana*, with control samples forming a separate cluster from all exposed groups (Fig 3A). Among neem-treated juveniles, the 100 and 200 ppm groups clustered together, whereas the 300 and 400 ppm groups showed partially overlapping but distinct profiles.

**Fig 3:**
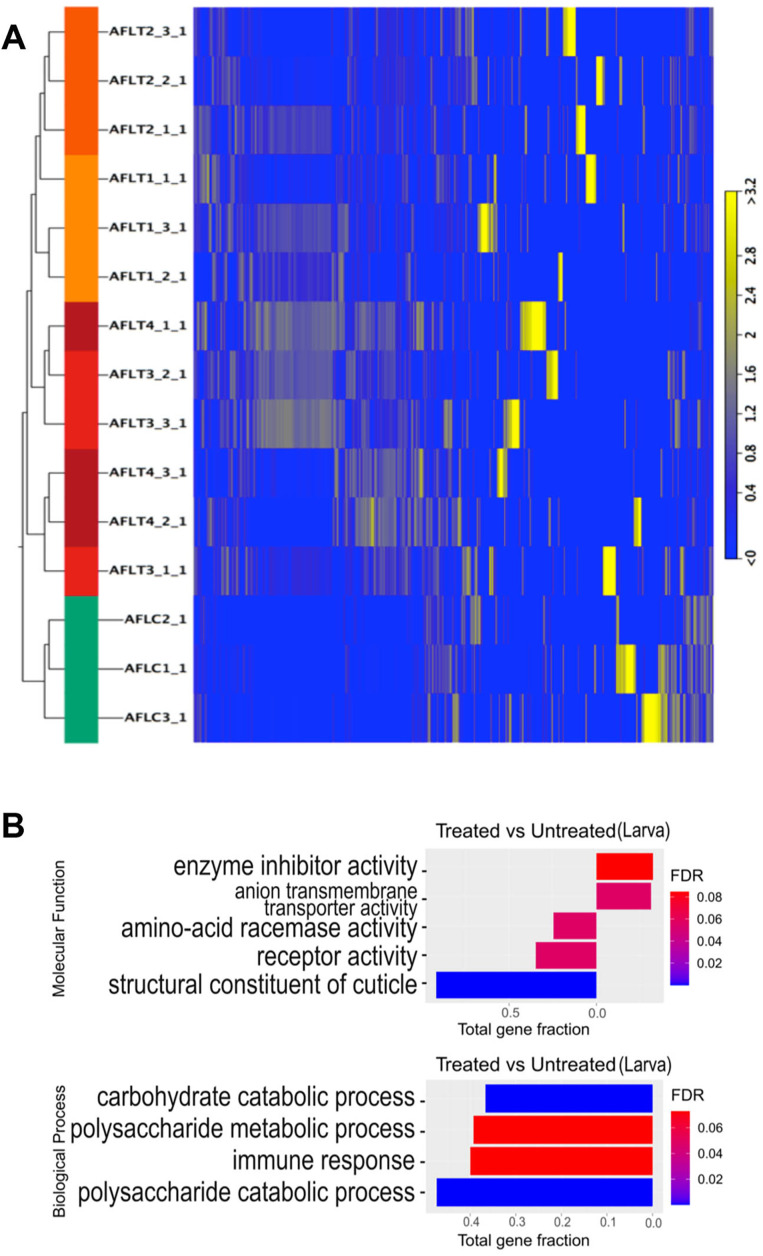
Transcriptomic responses to neem extract exposure in juvenile *A. franciscana.* **(A)** Heat map of hierarchical clustering of differentially expressed transcripts from juvenile *A. franciscana* exposed for 24 h to 0 (control) or 100, 200, 300 and 400 ppm neem (n = 3 libraries per treatment; 15 libraries in total). Columns represent individual libraries (controls in green; neem-treated groups in progressively darker warm colours from 100 to 400 ppm). The colour scale indicates relative transcript abundance, from lower (blue) to higher (yellow) expression. **(B)** Enriched GO categories for molecular function in treated (left) and untreated (right) juveniles. Bar length indicates the number of differentially-expressed transcripts assigned to each GO term. “Total gene fraction” denotes the proportion of all annotated transcripts in the transcriptome associated with that term. Only terms with P < 0.01 are shown. The colour gradient reflects false discovery rate (FDR), with blue indicating lower FDR (higher confidence) and red indicating higher FDR.

Differential expression analysis (Table 2) identified a set of candidate contigs with pronounced changes in expression following neem exposure. Strongly induced genes included *pseudouridine-5′-phosphatase* (log₂FC = 3.04), an enzyme involved in RNA modification, and *GTP cyclohydrolase 1* (log₂FC = 2.99), which participates in folate biosynthesis. Other upregulated transcripts included *cathepsin S* (log₂FC = 2.97), a lysosomal protease associated with protein catabolism, and *mitochondrial uncoupling protein 3* (log₂FC = 2.87), a protein linked to regulation of mitochondrial membrane potential.

**Table 2:** Differentially expressed transcripts in juvenile *Artemia franciscana* exposed for 24 h to neem extract, compared with untreated controls. The table lists the top 10 upregulated and top 10 downregulated transcripts, ranked by |log₂ fold change|. For each contig, UniProt accession, lowest BLASTx E-value, FDR-adjusted p-value and log₂ fold change are shown together with the corresponding protein annotation.

JUVENILES					
*Upregulated genes*					
Name	UniProt accession number	Lowest E-value	FDR p-value	Log2 fold change	Protein name
**contig_ 0015430**	Q08623	5,68E-69	1,29E-05	3,0369	Pseudouridine-5’-phosphatase
**contig_ 0087730**	Q94465	3,03E-54	8,15E-05	2,9936	GTP cyclohydrolase 1
**contig_ 0046175**	Q8HY81	2,69E-59	0,0001	2,9658	Cathepsin S
**contig_ 0023314**	Q94529	3,82E-78	0,0002	2,9013	Probable pseudouridine-5’-phosphatase
**contig_ 0035087**	O97649	9,23E-89	0,0002	2,8655	Mitochondrial uncoupling protein 3
**contig_ 0014471**	P17720	3,15E-131	0,0007	2,7867	Artemin
**contig_ 0019983**	P20073	2,25E-87	0	2,7626	Annexin A7
**contig_ 0038865**	Q5R5A8	3,31E-114	1,11E-05	2,6994	Mitochondrial uncoupling protein 2
**contig_ 0026028**	Q3U481	4,59E-79	0,00016	2,6682	Major facilitator superfamily domain-containing protein 12
**contig_ 0066299**	Q9KQQ6	0	0,01581	2,5536	Bifunctional protein FolD
					
***Downregulated genes***					
**contig_ 0034035**	P35502	1,33E-54	0	−4,2392	Esterase FE4
**contig_ 0014694**	Q8R0W5	2,31E-56	3,73E-13	−3,7970	Carboxylesterase 4A
**contig_ 0014205**	Q9NUB1	0	0	−3,5590	Acetyl-coenzyme A synthetase 2-like
**contig_ 0035915**	B4JG39	2,46E-52	0	−3,4898	Sensory neuron membrane protein 1
**contig_ 0019790**	A0A452E9Y6	7,86E-67	0	−3,2301	Lactoperoxidase
**contig_ 0012068**	P54316	2,89E-70	0	−3,2233	Inactive pancreatic lipase-related protein 1
**contig_ 0015718**	P04069	1,09E-99	6,30E-12	−3,2027	Carboxypeptidase B
**contig_ 0020784**	P35502	4,01E-55	2,52E-06	−3,0490	Esterase FE4
**contig_ 0000429**	Q9VEG6	1,61E-104	0	−3,0242	Chorion peroxidase
**contig_ 0003587**	Q00871	6,52E-85	9,88E-12	−2,8792	Chymotrypsin BI

Several transcripts encoding enzymes related to metabolism and detoxification showed marked reductions in expression levels. These included *esterase FE4* (log₂FC = −4.24) and *carboxylesterase 4A* (log₂FC = −3.80), both esterases commonly associated with xenobiotic processing, as well as an *acetyl-CoA synthetase 2-like* enzyme (log₂FC = −3.56), involved in acyl-CoA formation. Transcripts annotated as *lactoperoxidase* (log₂FC = −3.23), a heme-containing oxidoreductase, and a *pancreatic lipase-related protein* (log₂FC = −3.22), implicated in lipid hydrolysis, were also significantly downregulated in neem-treated juveniles.

Gene Ontology (GO) enrichment analysis in juveniles (Fig 3B) showed significant over-representation of metabolic, immune, and structural functional categories. For biological processes, carbohydrate and polysaccharide catabolic processes were among the most strongly enriched terms. Immune-related categories were also enriched, including terms associated with innate and stress-related immune responses. For molecular function, the term “structural constituent of cuticle” showed the highest enrichment, reflecting consistent changes in genes encoding cuticle-associated proteins.

### 3.5. Targeted analysis of chitin-related transcripts in juvenile *A. franciscana*

In addition to the global transcript-level analysis, we inspected the abundance of a predefined panel of eight candidate contigs annotated as related to chitin precursor metabolism and chitin-associated processes in juvenile *A. franciscana*. This analysis was based on TPM-derived abundance estimates from the same RNA-seq dataset and is therefore interpreted as an exploratory candidate-transcript analysis. Seven of these genes were significantly downregulated in neem-treated juveniles compared with controls. The strongest reductions were observed for *hexose phosphate aminotransferase 1* and *chitin synthase chs2* (Fig 4A,4B), which encode enzymes involved in precursor production and chitin polymerization, respectively. Overall, neem-treated juveniles showed lower transcript abundance for several chitin-pathway components during early developmental stages.

**Fig 4:**
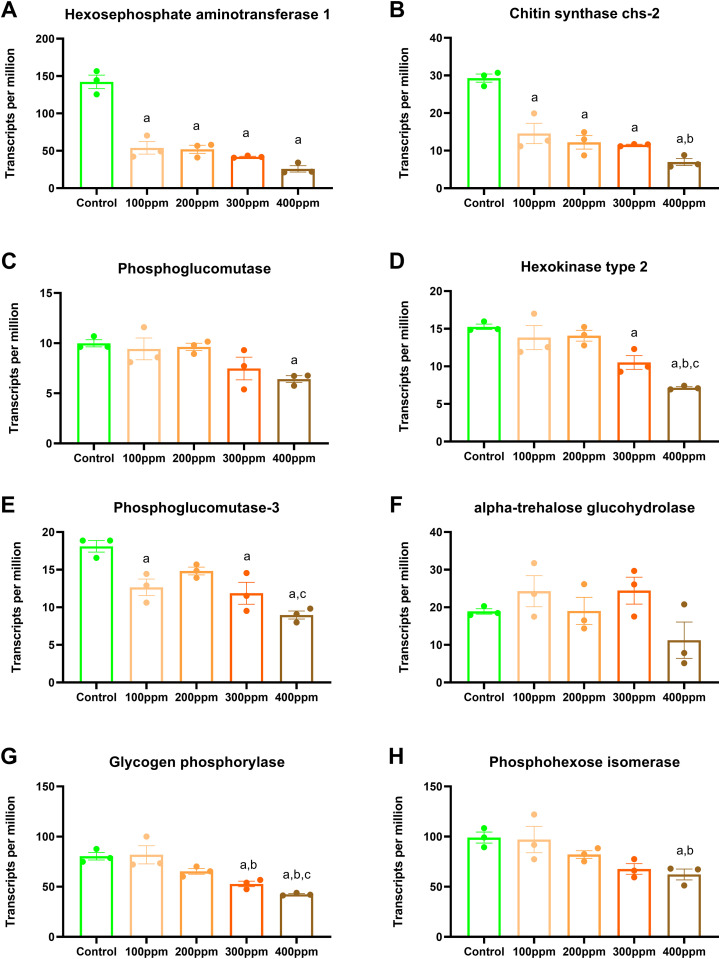
Expression of chitin-related transcripts in juvenile *A. franciscana* exposed to neem extract. Transcripts per million (TPM) for selected contigs involved in chitin precursor production and chitin polymerization in juveniles exposed for 24 h to 0, 100, 200, 300, and 400 ppm neem extract. Panels show (A) *hexosephosphate aminotransferase 1*, (B) *chitin synthase chs-2*, (C) *phosphoglucomutase*, (D) *hexokinase type 2*, (E) *phosphoglucomutase-3*, (F) *alpha-trehalose glucohydrolase*, (G) *glycogen phosphorylase*, and (H) *phosphohexose isomerase*. Bars show mean ± SEM (n = 3 libraries per treatment). Different lowercase letters indicate significant differences among treatments (one-way ANOVA followed by Tukey’s multiple comparison test).

### 3.6. Adult transcript abundance patterns indicate stress-, metabolism-, and structural candidate responses to neem exposure

Exposure to neem extract was associated with transcript abundance differences in adult *A. franciscana*. Hierarchical clustering of differentially-expressed transcripts showed that untreated samples generally formed a distinct cluster from treated groups (Fig 5A). One sample from the 500 ppm treatment (AFATT2_1_1) grouped with controls, indicating within-group variation. Despite this, most treated samples exhibited transcriptional shifts compared to controls. Within the treated groups, adults exposed to 500 ppm displayed a transcriptional profile that differed from both the 250 ppm group and untreated controls.

**Fig 5:**
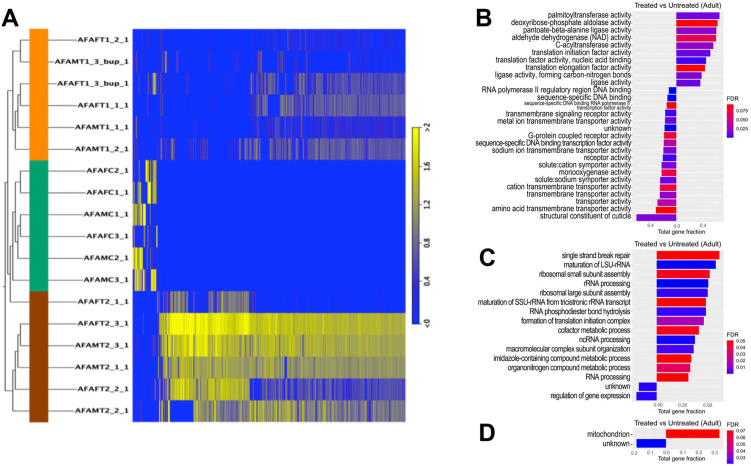
Transcriptomic responses to neem extract exposure in adult *A. franciscana.* **(A)** Heat map of hierarchical clustering of differentially-expressed transcripts from adult *A. franciscana* exposed for 7 days to 0 (control), 250 and 500 ppm neem (n = 3 libraries per sex and treatment). Colours indicate relative transcript abundance (low to high). **(B–D)** Enriched Gene Ontology (GO) categories for molecular function **(B)**, biological process **(C)** and cellular component **(D)** in treated (left) and untreated (right) adults. Bar length indicates the number of DEGs assigned to each GO term. “Total gene fraction” denotes the proportion of all annotated contigs in the transcriptome associated with that term. Only terms with P < 0.01 are shown. The colour gradient reflects false discovery rate (FDR), with blue indicating lower FDR (higher confidence) and red indicating higher FDR.

Differential expression analysis (Table 3) identified several transcripts with large changes in expression in neem-exposed adults. Strongly upregulated transcripts included *elongation factor 1-alpha* (log₂FC = 14.09), a translation factor involved in protein synthesis, and *tubulin alpha chain* (log₂FC = 13.54), a major component of the cytoskeleton. *Heat shock protein 70 protein 4 (HSPA4)* (log₂FC = 12.07), a molecular chaperone of the HSP70 family, was also highly induced, as was a *major cysteine proteinase* (log₂FC = 13.18), a protease associated with protein turnover.

**Table 3:** Differentially-expressed transcripts in adult *Artemia franciscana* exposed for 7 days to neem extract, compared with untreated controls. The table lists the top 10 upregulated and top 10 downregulated transcripts, ranked by |log₂ fold change|. For each contig, UniProt accession, lowest BLASTx E-value, FDR-adjusted p-value and log₂ fold change are shown together with the corresponding protein annotation.

ADULTS					
Upregulated genes					
Name	UniProt accession number	Lowest E-value	FDR p-value	Log2 fold change	Protein name
contig_ 0005285	P90519	9.95E-116	3.54E-11	14.0898	Elongation factor 1-alpha
contig_ 0030322	P04106	0	1.19E-10	13.5367	Tubulin alpha chain
contig_ 0036860	P25779	3.73E-113	6.10E-10	13.1809	Major cysteine proteinase
contig_ 0017701	A5DI11	0	8.40E-09	12.4030	Elongation factor 2
contig_ 0064049	P31692	3.40E-84	1.62E-08	12.1311	Adenine nucleotide translocator
contig_ 0022295	P11145	5.99E-99	2.97E-08	12.0665	Heat shock 70 kDa protein 4
contig_ 0026696	P04106	0	8.02E-08	11.9981	Tubulin alpha chain
contig_ 0071908	P26796	3.27E-122	3.23E-08	11.8823	60S acidic ribosomal protein P0
contig_ 0043702	O13672	2.50E-60	4.99E-08	11.8531	60S ribosomal protein L8
contig_ 0039289	P69104	1.16E-122	4.75E-08	11.8479	Guanine nucleotide-binding protein subunit beta-like protein
**Downregulated genes**					
contig_ 0055157	Q9JME2	7.25E-61	1.34E-08	−6.6510	Carbohydrate sulfotransferase 11
contig_ 0086110	P13706	1.46E-53	0.00717	−4.8626	Glycerol-3-phosphate dehydrogenase [NAD(+)], cytoplasmic
contig_ 0022053	P00765	5.47E-51	4.02E-05	−3.0847	Trypsin-1
contig_ 0005802	P00771	2.96E-53	0.00063	−2.9908	Collagenolytic protease
contig_ 0032565	Q9S777	7.65E-65	9.71E-05	−2.9622	4-coumarate--CoA ligase 3
contig_ 0002900	P09470	8.25E-82	0.00512	−2.7686	Angiotensin-converting enzyme
contig_ 0015718	P04069	1.09E-99	2.20E-05	−2.7198	Carboxypeptidase B
contig_ 0014694	Q8R0W5	2.31E-56	8.90E-08	−2.6582	Carboxylesterase 4A
contig_ 0057631	P27619	2.12E-98	0.03036	−2.6305	Dynamin
contig_ 0000432	Q04960	2.13E-95	0.00055	−2.5258	DnaJ protein homolog

Several transcripts associated with energy metabolism and other homeostatic functions showed reduced expression levels in adults exposed to neem. *Glycerol-3-phosphate dehydrogenase*, an enzyme involved in glycolysis and lipid metabolism, was strongly downregulated (log₂FC = −4.86), as was *carbohydrate sulfotransferase 11* (log₂FC = −6.65), which participates in glycosaminoglycan biosynthesis.

Gene Ontology (GO) enrichment analysis in adults (Fig 5B–5D) showed significant over-representation of categories related to “regulation of gene expression” and “transcription factor activity” in treated samples. In contrast, untreated adults exhibited higher representation of GO terms such as “ribosomal large subunit assembly” and “rRNA processing”, as well as lipid metabolic processes including lipase and aldehyde dehydrogenase activities. Treated adults also showed enrichment of structural molecule activity associated with the extracellular region and cuticle.

Collectively, these transcript abundance and GO patterns indicate candidate stress-, metabolism- and cuticle-associated responses to neem exposure in adults.

### 3.7. Exploration of candidate chitin-related transcript expression in adult *A. franciscana* exposed to neem

To determine whether chitin-associated pathways were also affected in adults, we analysed the same panel of eight genes related to chitin biosynthesis and degradation used for juveniles in neem-treated and untreated adult *A. franciscana* (Fig 6). Three of these transcripts showed significant expression differences between treatments. In males, *hexokinase type 2* and *phosphohexose isomerase*, both associated with carbohydrate metabolism and chitin precursor synthesis, were significantly downregulated in neem-exposed individuals compared with controls (Fig 6A,6B). In females, *glycogen phosphorylase*, an enzyme contributing to mobilization of stored carbohydrates toward biosynthetic pathways, showed reduced expression in treated samples (Fig 6C).

**Fig 6:**
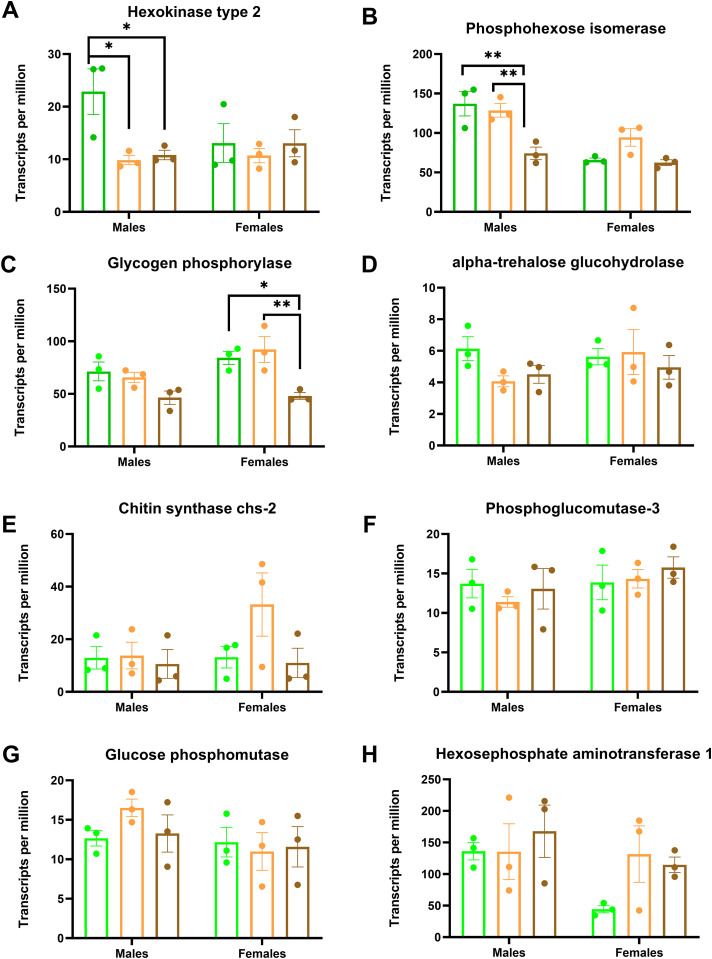
Expression of chitin-related transcripts in adult *A. franciscana* exposed to neem extract. Transcripts per million (TPM) for selected contigs involved in chitin precursor metabolism in adult males and females exposed for 7 days to 0, 250, and 500 ppm neem extract. Panels show (A) *hexokinase type 2*, (B) *phosphohexose isomerase*, (C) *glycogen phosphorylase*, (D) *alpha-trehalose glucohydrolase*, (E) *chitin synthase chs-2*, (F) *phosphoglucomutase-3*, (G) *glucose phosphomutase*, and (H) *hexosephosphate aminotransferase 1*. Bars show mean ± SEM (n = 3 libraries per sex and treatment). Brackets indicate the pairwise comparisons tested, and asterisks indicate statistically significant differences between groups (*P < 0.05, **P < 0.01; two-way ANOVA followed by Tukey’s multiple comparison test).

### 3.8. Histological analysis of adult *Artemia*

Histological analysis was used to evaluate reproductive tissue morphology in adult *A. franciscana* exposed to neem extract. In untreated control females (0 ppm; Fig 7A–7C), the embryo-bearing brood sac region showed a defined cuticular boundary enclosing the embryos; the moon-shaped embryos within the brood sac corresponded to desiccating *A. franciscana* cysts (arrows) and exhibited normal morphology. In females exposed to 100 and 250 ppm neem extract (Fig 7D–7I), representative sections showed altered organization of this region, including irregular contour of the cuticular boundary and less sharply delimited embryo-associated structures relative to controls. In females exposed to 500 ppm (Fig 7J–7L), the embryo-bearing brood sac region showed more evident loss of structural organization, including discontinuous or poorly delimited areas of the cuticular boundary and irregular arrangement of embryo-associated structures. Cuticle thickness was not quantitatively assessed in this analysis.

**Fig 7:**
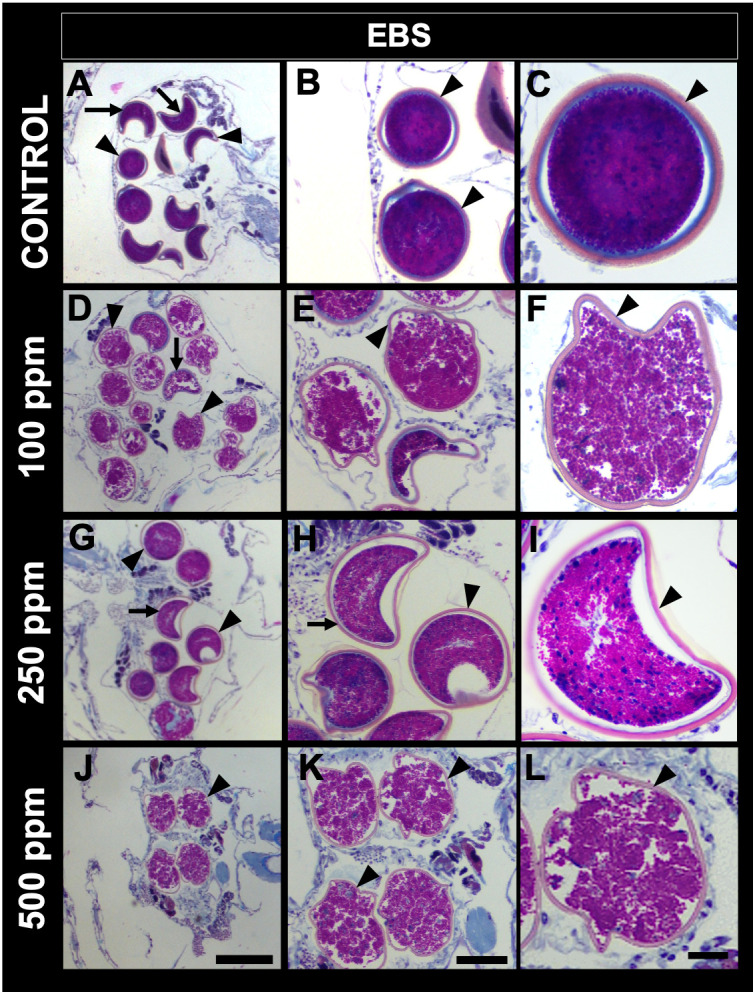
Histological sections of the embryo-bearing brood sac region in the adult female reproductive tract of *Artemia franciscana.* Arrowheads indicate the cuticular boundary surrounding embryo-associated structures in the brood sac region. Arrows indicate desiccating *Artemia* cysts within the brood sac. (A–C) Untreated control female. (D–F) Female exposed to 100 ppm neem extract. (G–I) Female exposed to 250 ppm neem extract. (J–L) Female exposed to 500 ppm neem extract. Representative treated sections show altered organization of the embryo-bearing brood sac region, including irregular or less sharply delimited areas of the cuticular boundary and embryo-associated structures. Moon-shaped structures within the brood sac correspond to *Artemia* cysts in the process of desiccation. (A, D, G, J): Bar = 200 µm. (B, E, H, K): Bar = 100 µm. (C, F, I, L): Bar = 50 µm.

Female ovarian area was quantified as a histometric endpoint, whereas direct reproductive output, including released cysts or nauplii, was not measured. Beyond the brood sac region, morphometric analysis showed that females from higher neem treatments had a significantly reduced ovarian area (P = 0.0006) compared to controls (S1 Fig). In contrast, males did not show significant changes in testis area. The gonadal area measurements used to generate S1 Fig are provided in S1 Dataset.

## 4. Discussion

Neem exposure produced strong, dose-dependent effects in *A. franciscana*, affecting both juveniles and adults. Larvae exhibited high mortality and abnormal phenotypes at concentrations ≥300 ppm, while adults displayed significant mortality after 7-day exposures to 500 ppm. Males were more susceptible than females, a pattern consistent with documented sex-specific sensitivities to toxicants in crustaceans [30–33]. Baseline transcriptomic studies have shown that males and females of *A. franciscana* differ markedly in gene expression profiles [34,35]. Together with our transcriptomic and histological data, these phenotypic outcomes indicate that neem-derived compounds were associated with marked changes in survival, development, and reproductive-associated endpoints in this model crustacean.

Transcriptomic analysis suggested life stage- and sex-specific transcript abundance patterns associated with neem exposure. While both juveniles and adults mounted transcriptomic responses, adults exhibited more extensive changes (19,957 differentially expressed contigs vs. 933 in juveniles), suggesting heightened transcriptional reprogramming. Such widespread changes reflect the activation of stress response pathways and suppression of metabolic processes, consistent with toxicant-induced physiological disruption in other aquatic arthropods [36–38]. A comparable pattern has been described in insect larvae exposed to azadirachtin, where large-scale transcriptomic reprogramming affected more than 1,200 genes in *Spodoptera frugiperda*, including detoxification and structural genes, together with suppression of chitin synthase and other chitin- and cuticle-associated genes involved in cuticle formation [39]. In line with these findings, one of the most prominent transcriptomic signatures in juvenile *A. franciscana* was the disruption of chitin metabolism, with seven of eight analyzed chitin-related transcripts significantly downregulated, including *hexose phosphate aminotransferase* and *chs2*, which are critical for chitin precursor production and polymerization during exoskeleton formation. Adults also exhibited significant transcriptomic disruptions, particularly upregulation of heat shock proteins (e.g., HSPA4), tubulin, and cysteine proteases, indicating activation of stress, cytoskeletal reorganization, and tissue remodeling responses. Downregulated transcripts were enriched for energy metabolism and protein biosynthesis functions, including glycolytic enzymes, sulfotransferases, and ribosomal proteins. These patterns suggest a shift from growth-related processes to cellular maintenance and survival—a known stress response in arthropods [40,41].

A limitation of this study is that the RNA-seq analysis relied on a de novo assembled transcriptome rather than genome-guided quantification. Recent chromosome-level genomic resources are available for *A. franciscana* [42], and their use in future analyses would improve gene-level resolution, orthology-aware annotation, and comparability with genomic resources. Consequently, the transcriptomic results reported here should be interpreted as treatment-associated transcript/contig abundance patterns and candidate functional responses, rather than definitive gene-level regulatory effects. The targeted analysis of candidate chitin-related transcripts provides a focused transcriptomic context for interpreting the cuticle- and carbohydrate-associated responses observed after neem exposure. These contigs were selected from the annotated transcriptome based on their functional association with chitin precursor metabolism and cuticle-related pathways, and their abundance patterns were consistent with the broader differential-abundance and GO enrichment results. However, because this analysis was based on TPM-derived RNA-seq estimates from the same dataset, it should be considered a transcriptomic extension of the global analysis rather than an independent functional validation. Future studies incorporating RT-qPCR, chitin synthase or chitinase activity assays, and direct biochemical or histochemical measurements of chitin content would help determine whether these transcript-level responses translate into functional changes in chitin metabolism or cuticle formation.

Expression of chitin-related contigs in adults showed selective downregulation of enzymes involved in carbohydrate metabolism, notably in males. Reduced expression of *hexokinase type 2* and *phosphohexose isomerase* in males, and *glycogen phosphorylase* in females, is consistent with reduced capacity for UDP-N-acetylglucosamine production, the key precursor for chitin biosynthesis. These enzymes participate in the early steps of the hexosamine pathway supplying substrates for chitin formation [43–45], and glycogen phosphorylase is known to mobilize stored carbohydrates into this biosynthetic route [44,46]. Since chitin synthesis underpins the structural integrity of crustacean exoskeletal and reproductive tissues, including brood sac and embryonic envelopes [47,48], we speculate that these molecular alterations may contribute to the brood sac changes observed histologically.

Histological analysis provided morphological evidence of treatment-associated alterations in the female reproductive region. In treated females, the embryo-bearing brood sac region showed altered organization, including irregular contours of the cuticular boundary and poorly delimited embryo-associated structures, while morphometric analysis showed a significant reduction in ovarian area. These observations are consistent with the transcript abundance patterns identified in the transcriptomic analysis, particularly those involving cuticle-associated and chitin-related candidate transcripts. However, because cuticle thickness was not quantitatively assessed, the histological data should not be interpreted as direct evidence of measured cuticle thinning or altered chitin deposition. Similarly, because direct reproductive output was not measured, including released cysts or nauplii, reduced ovarian area should be interpreted as a treatment-associated histometric change in reproductive tissue condition rather than as direct evidence of reduced reproductive performance. Although ovarian size in *Artemia* females may vary according to reproductive stage [49], which limits its interpretation as a stand-alone reproductive endpoint, reproductive impairments have been reported in other invertebrates exposed to neem-based products. In *Daphnia*, chronic neem exposure reduced offspring viability [50], while in *Bombus terrestris*, azadirachtin ingestion caused ovary atrophy and cessation of egg-laying [51]. Together, these findings support the hypothesis that neem exposure affects reproductive-associated tissues in *A. franciscana*, but future studies should include direct counts of released cysts and nauplii, quantitative cuticle measurements, and functional assays of chitin metabolism to establish the reproductive and structural consequences of these changes.

An additional limitation concerns the chemical characterization of the exposure solution. The compositional values reported for the neem kernel extract correspond to the manufacturer’s specification for the commercial product, and the concentrations tested here were nominal concentrations of total neem extract rather than analytically confirmed concentrations of azadirachtin A, additional azadirachtins, or individual limonoids. Therefore, the toxicological responses reported in this study should be interpreted as responses to the tested commercial neem kernel extract formulation, not to purified azadirachtin A or to a chemically standardized limonoid mixture. From a formulation and chemistry perspective, it is also important to note the compositional distinctions between neem kernel and neem oil. Neem kernel is rich in azadirachtin and related limonoids, while neem oil contains primarily fatty acids and acts as a delivery enhancer. The extract used in our study derived from kernel content, where azadirachtin is a dominant component known to inhibit chitin synthesis and disrupt molting [52,53]. Although this was not directly tested here**,** the potential synergy between oil-based lipids and kernel-derived limonoids may amplify toxicological outcomes in certain contexts [54,55]. Understanding these chemical distinctions is essential for interpreting variability across studies and guiding safe application in aquaculture settings.

Although neem is often perceived as an environmentally benign biopesticide, several studies have shown that neem-based formulations can exert lethal and sublethal effects on non-target aquatic organisms, including invertebrates and fish [8,50,56,57]. Our results add to this evidence by showing that, under the laboratory conditions used here, neem exposure was associated with phenotypic abnormalities, histological alterations, and transcriptomic changes in *A. franciscana*. Given that zooplanktonic crustaceans play central roles in many aquatic food webs, comparable sublethal effects in ecologically relevant taxa could, in principle, translate into altered population dynamics and ecosystem functioning [17,18]. The combined use of transcriptomic and histological endpoints in the present study illustrates how mechanistic biomarkers can reveal early signs of stress and toxicity before overt population-level declines become apparent and supports their broader application in ecotoxicological risk assessment [9,13,58].

Finally, this study highlights the utility of combining high-throughput transcriptomics with classical ecotoxicological endpoints. Gene expression profiling provided molecular information on stress, detoxification, and structural impairments that are not discernible from phenotypic observations alone, consistent with the growing use of omics-based tools in environmental monitoring and pesticide risk assessment [9,10]. In summary, neem extract was associated with changes in *A. franciscana* related to exoskeletal maintenance, metabolism, and reproduction, together with transcriptomic and histological alterations. These observations are relevant to ongoing efforts to integrate molecular data into structured ecotoxicological interpretation frameworks, including Adverse Outcome Pathway–based approaches [59]. Together, these results contribute to understanding of how botanical insecticides affect non-target aquatic invertebrates and support careful evaluation of their use in habitats supporting sensitive species.

## Supporting information

S1 Table cDNA libraries included in the RNA-seq experiment for juvenile and adult *Artemia franciscana.* For each library, sample name, developmental stage, sex, treatment condition, and neem concentration are indicated.
(DOCX)

S1 Dataset Raw numerical data underlying the phenotypic, survival, and histometric analyses. This workbook contains the raw numerical data used to generate Fig 1A–C and S1 Fig. The dataset includes replicate-level juvenile *Artemia franciscana*phenotype counts for the 24 h exposure assay shown in Fig 1A, adult survival data by sex, treatment, and day for Fig 1B–C, and individual-level ovarian and testis area measurements used for S1 Fig.
(XLSX)

S1 Video Representative stereomicroscope recordings of juvenile (naupliar-stage) *Artemia franciscana* exposed for 24 h to neem extract. On-screen concentration labels are expressed in g/L: 0 (control), 0.1, 0.2, 0.3, 0.4, 0.5, 0.6, 0.7, and 0.8 g/L, corresponding to 0, 100, 200, 300, 400, 500, 600, 700, and 800 mg/L, respectively. Normal juveniles show active swimming with coordinated, undulating movements of the podia and maintain their position in the water column. Abnormal juveniles display reduced and/or uncoordinated podial movements and are unable to maintain their position in the water column. Dead juveniles remain immobile on the well bottom, show no response to gentle tapping, and may exhibit necrotic morphology.
(MP4)

S2 Video Reference video for adult sex identification in control *Artemia franciscana.* Representative stereomicroscope recordings of unexposed adult A. franciscana of both sexes used for the 7-day survival experiment. Males are identified by the presence of claspers, and females are shown as gravid (ovoviviparous).
(MP4)

S1 Fig Gonadal area of adult *Artemia franciscana* exposed to neem extract. Gonadal area of females and males after 7 days of exposure to 0, 100, 250, and 500 mg/L neem extract. (A) Ovarian area in females. (B) Testis area in males. Data are shown as mean ± SD (n = 14 individuals per sex and treatment). Asterisks indicate significant differences from the corresponding control group (one-way ANOVA followed by Tukey’s multiple comparison test, P < 0.05); no significant differences were detected among male treatments.
(TIF)

## References

[1] Adusei S, Azupio S. Neem: a novel biocide for pest and disease control of plants. Journal of Chemistry. 2022;2022:1–12. doi: 10.1155/2022/6778554

[2] Isman MB. Botanical insecticides in the twenty-first century—fulfilling their promise?. Annu Rev Entomol. 2020;65(1):233–49. doi: 10.1146/annurev-ento-011019-025010 31594414

[3] Kilani-Morakchi S, Morakchi-Goudjil H, Sifi K. Azadirachtin-based insecticide: Overview, risk assessments, and future directions. Front Agron. 2021;3:676208. doi: 10.3389/fagro.2021.676208

[4] Aly SM, Abou El-Gheit SN, Essam El-Din HM. Comparative studies on the efficiency of neem leaves azadirachta indica and flubendazole treatment against diplectanum in sea bass dicentrarchus labrax. Acta Parasitol. 2022;67(2):970–5. doi: 10.1007/s11686-022-00544-2 35426620 PMC9165231

[5] Ibrahim RE, Elshopakey GE, Abdelwarith AA, Younis EM, Ismail SH, Ahmed AI, et al. Chitosan neem nanocapsule enhances immunity and disease resistance in nile tilapia (Oreochromis niloticus). Heliyon. 2023;9(9):e19354. doi: 10.1016/j.heliyon.2023.e19354 37662722 PMC10474430

[6] Kim KS, Walker GC. Efficacy of neem extract against sea lice (Lepeophtheirus salmonis) infestations: a potential biopesticide for Atlantic salmon (Salmo salar). Aquaculture. 2022;560:738453. doi: 10.1016/j.aquaculture.2022.738453

[7] Talpur AD, Ikhwanuddin M. Azadirachta indica (neem) leaf dietary effects on the immunity response and disease resistance of Asian seabass, Lates calcarifer challenged with Vibrio harveyi. Fish Shellfish Immunol. 2013;34(1):254–64. doi: 10.1016/j.fsi.2012.11.003 23178500

[8] Sacco D, Velíšek J, Mikušková N. Toxicological effects of azadirachtin on aquatic species: a review of its role in biopesticides. Aquat Toxicol. 2025;288:107547. doi: 10.1016/j.aquatox.2025.107547 40897009

[9] Bakker R, Ellers J, Roelofs D, Vooijs R, Dijkstra T, van Gestel CAM, et al. Combining time-resolved transcriptomics and proteomics data for adverse outcome pathway refinement in ecotoxicology. Sci Total Environ. 2023;869:161740. doi: 10.1016/j.scitotenv.2023.161740 36708843

[10] Caputo DR, Robson SC, Werner I, Ford AT. Complete transcriptome assembly and annotation of a critically important amphipod species in freshwater ecotoxicological risk assessment: Gammarus fossarum. Environ Int. 2020;137:105319. doi: 10.1016/j.envint.2019.105319 32028177

[11] Péden R, Poupin P, Sohm B, Flayac J, Giambérini L, Klopp C, et al. Environmental transcriptomes of invasive dreissena, a model species in ecotoxicology and invasion biology. Sci Data. 2019;6(1):234. doi: 10.1038/s41597-019-0252-x 31653851 PMC6814772

[12] Falciani F, Diab AM, Sabine V, Williams TD, Ortega F, George SG, et al. Hepatic transcriptomic profiles of European flounder (Platichthys flesus) from field sites and computational approaches to predict site from stress gene responses following exposure to model toxicants. Aquat Toxicol. 2008;90(2):92–101. doi: 10.1016/j.aquatox.2008.07.020 18823667

[13] Garcia-Reyero N, Perkins EJ. Systems biology: leading the revolution in ecotoxicology. Environ Toxicol Chem. 2011;30(2):265–73. doi: 10.1002/etc.401 21072840

[14] Ankley GT, Bennett RS, Erickson RJ, Hoff DJ, Hornung MW, Johnson RD, et al. Adverse outcome pathways: a conceptual framework to support ecotoxicology research and risk assessment. Environ Toxicol Chem. 2010;29(3):730–41. doi: 10.1002/etc.34 20821501

[15] OECD. Users’ handbook supplement to the guidance document for developing and assessing adverse outcome pathways. 2018.

[16] Bergami E, Bocci E, Vannuccini ML, Monopoli M, Salvati A, Dawson KA, et al. Nano-sized polystyrene affects feeding, behavior and physiology of brine shrimp Artemia franciscana larvae. Ecotoxicol Environ Saf. 2016;123:18–25. doi: 10.1016/j.ecoenv.2015.09.021 26422775

[17] Brix KV, Cardwell RD, Adams WJ. Chronic toxicity of arsenic to the Great Salt Lake brine shrimp, Artemia franciscana. Ecotoxicol Environ Saf. 2003;54(2):169–75. doi: 10.1016/s0147-6513(02)00054-4 12550094

[18] Hadjispyrou S, Kungolos A, Anagnostopoulos A. Toxicity, bioaccumulation, and interactive effects of organotin, cadmium, and chromium on Artemia franciscana. Ecotoxicol Environ Safety. 2001;49(2):179–86. doi: 10.1006/eesa.2001.2059 11386732

[19] Sendra M, Sparaventi E, Blasco J, Moreno-Garrido I, Araujo CVM. Ingestion and bioaccumulation of polystyrene nanoplastics and their effects on the microalgal feeding of Artemia franciscana. Ecotoxicol Environ Saf. 2020;188:109853. doi: 10.1016/j.ecoenv.2019.109853 31704318

[20] Pan YJ, Dahms HU, Hwang JS, Souissi S. Recent trends in live feeds for marine larviculture: a mini review. Front Mar Sci. 2022;9:864165. doi: 10.3389/fmars.2022.864165

[21] Albarano L, Ruocco N, Lofrano G, Guida M, Libralato G. Genotoxicity in Artemia spp.: an old model with new sensitive endpoints. Aquat Toxicol. 2022;252:106320. doi: 10.1016/j.aquatox.2022.106320 36206704

[22] Libralato G, Prato E, Migliore L, Cicero AM, Manfra L. A review of toxicity testing protocols and endpoints with Artemia spp. Ecol Indicat. 2016;69:35–49. doi: 10.1016/j.ecolind.2016.04.017

[23] Goktepe I, Portier R, Ahmedna M. Ecological risk assessment of neem-based pesticides. J Environ Sci Health B. 2004;39(2):311–20. doi: 10.1081/pfc-120030244 15132337

[24] Jackson DJ, Cerveau N, Posnien N. De novo assembly of transcriptomes and differential gene expression analysis using short-read data from emerging model organisms - a brief guide. Front Zool. 2024;21(1):17. doi: 10.1186/s12983-024-00538-y 38902827 PMC11188175

[25] Raghavan V, Kraft L, Mesny F, Rigerte L. A simple guide to de novo transcriptome assembly and annotation. Brief Bioinform. 2022;23(2):bbab563. doi: 10.1093/bib/bbab563 35076693 PMC8921630

[26] Wright RM, Aglyamova GV, Meyer E, Matz MV. Gene expression associated with white syndromes in a reef building coral, Acropora hyacinthus. BMC Genomics. 2015;16(1):371. doi: 10.1186/s12864-015-1540-2 25956907 PMC4425862

[27] Fallet M, Montagnani C, Petton B, Dantan L, de Lorgeril J, Comarmond S, et al. Early life microbial exposures shape the Crassostrea gigas immune system for lifelong and intergenerational disease protection. Microbiome. 2022;10(1):85. doi: 10.1186/s40168-022-01280-5 35659369 PMC9167547

[28] Zhang X, Yuan J, Li F, Xiang J. Chitin synthesis and degradation in crustaceans: a genomic view and application. Mar Drugs. 2021;19(3):153. doi: 10.3390/md19030153 33804177 PMC8002005

[29] Westfall P, Henning KSS. Understanding advanced statistical methods. 1st ed. Chapman and Hall/CRC; 2013. doi: 10.1201/b14398

[30] Dong DT, Miranda AF, Carve M, Shen H, Trestrail C, Dinh KV, et al. Population- and sex-specific sensitivity of the marine amphipod Allorchestes compressa to metal exposure. Ecotoxicol Environ Saf. 2020;206:111130. doi: 10.1016/j.ecoenv.2020.111130 32866889

[31] Huang A, Roessink I, van den Brink NW, van den Brink PJ. Size- and sex-related sensitivity differences of aquatic crustaceans to imidacloprid. Ecotoxicol Environ Saf. 2022;242:113917. doi: 10.1016/j.ecoenv.2022.113917 35908530

[32] Kadiene EU, Bialais C, Ouddane B, Hwang J-S, Souissi S. Differences in lethal response between male and female calanoid copepods and life cycle traits to cadmium toxicity. Ecotoxicology. 2017;26(9):1227–39. doi: 10.1007/s10646-017-1848-6 28990129

[33] Wirth EF, Lund SA, Fulton MH, Scott GI. Determination of acute mortality in adults and sublethal embryo responses of Palaemonetes pugio to endosulfan and methoprene exposure. Aquat Toxicol. 2001;53(1):9–18. doi: 10.1016/s0166-445x(00)00157-0 11254943

[34] Huylmans AK, Toups MA, Macon A, Gammerdinger WJ, Vicoso B. Sex-biased gene expression and dosage compensation on the Artemia franciscana Z-chromosome. Genome Biology and Evolution. 2019;11(4):1033–44. doi: 10.1093/gbe/evz053 30865260 PMC6456005

[35] Valenzuela-Miranda D, Gallardo-Escárate C, Valenzuela-Muñoz V, Farlora R, Gajardo G. Sex-dependent transcriptome analysis and single nucleotide polymorphism (SNP) discovery in the brine shrimp Artemia franciscana. Mar Genomics. 2014;18PB:151–4. doi: 10.1016/j.margen.2014.10.007 25450167

[36] Gray BCT, Champion C, Broadhurst MK, Coleman MA, Benkendorff K. Effects of contaminants and flooding on the physiology of harvested estuarine decapod crustaceans: a global review and meta-analysis. Environ Pollut. 2025;364(Pt 2):125347. doi: 10.1016/j.envpol.2024.125347 39577610

[37] Jiang H, Li R, Zhao M, Peng X, Sun M, Liu C, et al. Toxic effects of combined exposure to cadmium and diclofenac on freshwater crayfish (Procambarus clarkii): insights from antioxidant enzyme activity, histopathology, and gut microbiome. Aquat Toxicol. 2024;268:106844. doi: 10.1016/j.aquatox.2024.106844 38295602

[38] Michalaki A, McGivern AR, Poschet G, Büttner M, Altenburger R, Grintzalis K. The effects of single and combined stressors on daphnids-enzyme markers of physiology and metabolomics validate the impact of pollution. Toxics. 2022;10(10):604. doi: 10.3390/toxics10100604 36287884 PMC9609890

[39] Shu B, Yu H, Li Y, Zhong H, Li X, Cao L, et al. Identification of azadirachtin responsive genes in Spodoptera frugiperda larvae based on RNA-seq. Pestic Biochem Physiol. 2021;172:104745. doi: 10.1016/j.pestbp.2020.104745 33518039

[40] Derecka K, Blythe MJ, Malla S, Genereux DP, Guffanti A, Pavan P, et al. Transient exposure to low levels of insecticide affects metabolic networks of honeybee larvae. PLoS One. 2013;8(7):e68191. doi: 10.1371/journal.pone.0068191 23844170 PMC3699529

[41] Kumar V, Roy S, Behera B, Das B. Heat Shock Proteins (Hsps) in cellular homeostasis: a promising tool for health management in crustacean aquaculture. Life. 2022;12(11):1777. doi: 10.3390/life12111777 36362932 PMC9699388

[42] Bett VK, Macon A, Vicoso B, Elkrewi M. Chromosome-level assembly of *Artemia franciscana* sheds light on sex chromosome differentiation. Genome Biol Evol. 2024;16(1):evae006. doi: 10.1093/gbe/evae006 38245839 PMC10827361

[43] Arakane Y, Muthukrishnan S. Insect chitinase and chitinase-like proteins. Cell Mol Life Sci. 2010;67(2):201–16. doi: 10.1007/s00018-009-0161-9 19816755 PMC11115512

[44] Chen QW, Jin S, Zhang L, Shen QD, Wei P, Wei ZM, et al. Regulatory functions of trehalose-6-phosphate synthase in the chitin biosynthesis pathway in *Tribolium castaneum* (Coleoptera: Tenebrionidae) revealed by RNA interference. Bull Entomol Res. 2018;108(3):388–99. doi: 10.1017/S000748531700089X 28920565

[45] Merzendorfer H. The cellular basis of chitin synthesis in fungi and insects: common principles and differences. Eur J Cell Biol. 2011;90(9):759–69. doi: 10.1016/j.ejcb.2011.04.014 21700357

[46] Tang B, Wei P, Zhao L, Shi Z, Shen Q, Yang M, et al. Knockdown of five trehalase genes using RNA interference regulates the gene expression of the chitin biosynthesis pathway in *Tribolium castaneum*. BMC Biotechnol. 2016;16(1):67. doi: 10.1186/s12896-016-0297-2 27596613 PMC5011928

[47] Ma W-M, Li H-W, Dai Z-M, Yang J-S, Yang F, Yang W-J. Chitin-binding proteins of Artemia diapause cysts participate in formation of the embryonic cuticle layer of cyst shells. Biochem J. 2013;449(1):285–94. doi: 10.1042/BJ20121259 23013449

[48] Younes I, Rinaudo M. Chitin and chitosan preparation from marine sources. Structure, properties and applications. Marine Drugs. 2015;13(3):1133–74. doi: 10.3390/md13031133 25738328 PMC4377977

[49] Duan H, Shao X, Liu W, Xiang J, Pan N, Wang X, et al. Spatio-temporal patterns of ovarian development and *VgR* gene silencing reduced fecundity in parthenogenetic *Artemia*. Open Biol. 2023;13(11):230172. doi: 10.1098/rsob.230172 37963545 PMC10645507

[50] Stark JD. Population-level effects of the neem insecticide, Neemix, on *Daphnia pulex*. J Environ Sci Health B. 2001;36(4):457–65. doi: 10.1081/PFC-100104189 11495023

[51] Barbosa WF, De Meyer L, Guedes RNC, Smagghe G. Lethal and sublethal effects of azadirachtin on the bumblebee Bombus terrestris (Hymenoptera: Apidae). Ecotoxicology. 2015;24(1):130–42. doi: 10.1007/s10646-014-1365-9 25300506

[52] Hazarika B, Saikia GK, Gogoi M, Bora D. Azadirachta indica biopesticide: A sustainable alternative to synthetic chemicals in pest control (a review). EEC. 2024;30:S271-6. doi: 10.53550/EEC.2024.v30i03s.047

[53] Soni P, Gupta P. Neem the source of versatile chemicals, Azadirachtin in modern pest management: a review. J Non-Timber Forest Prod. 2001;8(1/2):34–44. doi: 10.54207/bsmps2000-2001-v2986a

[54] Gauvin MJ, Bélanger A, Nébié R, Boivin G. Azadirachta indica: l’azadirachtine est-elle le seul ingrédient actif ?. Phyto. 2004;84(2):115–9. doi: 10.7202/007814ar

[55] Stark JD, Walter JF. Neem oil and neem oil components affect the efficacy of commercial neem insecticides. J Agric Food Chem. 1995;43(2):507–12. doi: 10.1021/jf00050a047

[56] Dunkel FV, Richards DC. Effect of an Azadirachtin Formulation on six nontarget aquatic macroinvertebrates. Environ Entomol. 1998;27(3):667–74. doi: 10.1093/ee/27.3.667

[57] Winkaler EU, Santos TRM, Machado-Neto JG, Martinez CBR. Acute lethal and sublethal effects of neem leaf extract on the neotropical freshwater fish Prochilodus lineatus. Comp Biochem Physiol C Toxicol Pharmacol. 2007;145(2):236–44. doi: 10.1016/j.cbpc.2006.12.009 17251062

[58] Revel M, Châtel A, Mouneyrac C. Omics tools: new challenges in aquatic nanotoxicology?. Aquatic Toxicology. 2017;193:72–85. doi: 10.1016/j.aquatox.2017.10.005 29049925

[59] Brockmeier EK, Hodges G, Hutchinson TH, Butler E, Hecker M, Tollefsen KE, et al. The role of omics in the application of adverse outcome pathways for chemical risk assessment. Toxicol Sci. 2017;158(2):252–62. doi: 10.1093/toxsci/kfx097 28525648 PMC5837273

