# Supplementary material for: Effects of neem extract on Artemia franciscana: Insights from high-throughput transcriptomics and phenotypic analysis
Source: PLoS One. 2026 Aug 11;21(8):e0348463. doi: 10.1371/journal.pone.0348463 (PMC13460601; doi:10.1371/journal.pone.0348463)
Supplement: Supplementary file 1 [file pone.0348463.s001.docx]

**S1 Table. cDNA libraries included in the RNA-seq experiment for juvenile and adult *Artemia franciscana*.** For each library, sample name, developmental stage, sex, challenge condition and neem dose are indicated.

| **Number** | **Sample name** | **Developmental Stage** | **Sex** | **Challenge** | **Neem** | **Dose (mg/L)** |
| --- | --- | --- | --- | --- | --- | --- |
| 1 | AFLC1 | Larvae | Undetermined | L_Control | Untreated | 0 |
| 2 | AFLC2 | Larvae | Undetermined | L_Control | Untreated | 0 |
| 3 | AFLC3 | Larvae | Undetermined | L_Control | Untreated | 0 |
| 4 | AFLT1_1 | Larvae | Undetermined | LT1 | Treated | 100 |
| 5 | AFLT1_2 | Larvae | Undetermined | LT1 | Treated | 100 |
| 6 | AFLT1_3 | Larvae | Undetermined | LT1 | Treated | 100 |
| 7 | AFLT2_1 | Larvae | Undetermined | LT2 | Treated | 200 |
| 8 | AFLT2_2 | Larvae | Undetermined | LT2 | Treated | 200 |
| 9 | AFLT2_3 | Larvae | Undetermined | LT2 | Treated | 200 |
| 10 | AFLT3_1 | Larvae | Undetermined | LT3 | Treated | 300 |
| 11 | AFLT3_2 | Larvae | Undetermined | LT3 | Treated | 300 |
| 12 | AFLT3_3 | Larvae | Undetermined | LT3 | Treated | 300 |
| 13 | AFLT4_1 | Larvae | Undetermined | LT4 | Treated | 400 |
| 14 | AFLT4_2 | Larvae | Undetermined | LT4 | Treated | 400 |
| 15 | AFLT4_3 | Larvae | Undetermined | LT4 | Treated | 400 |
| 16 | AFAMC1 | Adult | Male | A_Control | Untreated | 0 |
| 17 | AFAMC2 | Adult | Male | A_Control | Untreated | 0 |
| 18 | AFAMC3 | Adult | Male | A_Control | Untreated | 0 |
| 19 | AFAFC1 | Adult | Female | A_Control | Untreated | 0 |
| 20 | AFAFC2 | Adult | Female | A_Control | Untreated | 0 |
| 21 | AFAFC3 | Adult | Female | A_Control | Untreated | 0 |
| 22 | AFAMT1_1 | Adult | Male | AT1 | Treated | 250 |
| 23 | AFAMT1_2 | Adult | Male | AT1 | Treated | 250 |
| 24 | AFAMT1_3_bup | Adult | Male | AT1 | Treated | 250 |
| 25 | AFAFT1_1 | Adult | Female | AT1 | Treated | 250 |
| 26 | AFAFT1_2 | Adult | Female | AT1 | Treated | 250 |
| 27 | AFAFT1_3_bup | Adult | Female | AT1 | Treated | 250 |
| 28 | AFAMT2_1 | Adult | Male | AT2 | Treated | 500 |
| 29 | AFAMT2_2 | Adult | Male | AT2 | Treated | 500 |
| 30 | AFAMT2_3 | Adult | Male | AT2 | Treated | 500 |
| 31 | AFAFT2_1 | Adult | Female | AT2 | Treated | 500 |
| 32 | AFAFT2_2 | Adult | Female | AT2 | Treated | 500 |
| 33 | AFAFT2_3 | Adult | Female | AT2 | Treated | 500 |
